# Supplementary material for: Diverse Secondary Metabolites from the Coral-Derived Fungus Aspergillus hiratsukae SCSIO 5Bn1003
Source: Mar Drugs. 2022 Feb 18;20(2):150. doi: 10.3390/md20020150 (PMC8877224; doi:10.3390/md20020150)
Supplement: Supplementary file 1 [file marinedrugs-20-00150-s001.zip › marinedrugs-1597294-supplementary.pdf]

Diverse Secondary Metabolites from the Coral-Derived Fungus *Aspergillus hiratsukae* SCSIO 5Bn$_1$003

Qi Zeng [1,2], Yuchan Chen [3], Junfeng Wang [1], Xuefeng Shi [1], Yihao Che [1,2], Xiayu Chen [1,2], Weimao Zhong [1], Weimin Zhang [3], Xiaoyi Wei [4], Fazuo Wang [1,*] and Si Zhang [1,*]

[1] CAS Key Laboratory of Tropical Marine Bio-Resources and Ecology, Southern Marine Science and Engineering Guangdong Laboratory (Guangzhou), Guangdong Key Laboratory of Marine Materia Medica, RNAM Center for Marine Microbiology, South China Sea Institute of Oceanology, Chinese Academy of Sciences, 164 West Xingang Road, Guangzhou 510301, China; zengqi15@mails.ucas.ac.cn (Q.Z.); wangjunfeng@scsio.ac.cn (J.W.); shixuefeng@scsio.ac.cn (X.S.); cheyihao1995@163.com (Y.C.); chenxiayu17@mails.ucas.ac.cn (X.C.); wmzhong@scsio.ac.cn (W.Z.)

[2] University of Chinese Academy of Sciences, 19 Yuquan Road, Beijing 100049, China

[3] State Key Laboratory of Applied Microbiology Southern China, Guangdong Provincial Key Laboratory of Microbial Culture Collection and Application, Institute of Microbiology, Guangdong Academy of Sciences, 100 Central Xianlie Road, Guangzhou 510070, China; chenyc@gdim.cn (Y.C.); wmzhang@gdim.cn (W.Z.)

[4] Key Laboratory of Plant Resources Conservation and Sustainable Utilization, South China Botanical Garden, Chinese Academy of Sciences, Guangzhou 510650, China; wxy@scbg.ac.cn

* Correspondence: wangfazuo@scsio.ac.cn (F.W.); zhsimd@scsio.ac.cn (S.Z.)

List of Supporting Information

Figure S1. ^{1}H NMR Spectrum of **1**……………………………………………………3
Figure S2. ^{13}C and DEPT NMR Spectrum of **1**………………………………….……3
Figure S3. ^{1}H-^{1}H COSY Spectrum of **1**……………………………………………..…4
Figure S4. HSQC Spectrum of **1**…………………………………………………………4
Figure S5. HMBC Spectrum of **1**……………………………………………………...…5
Figure S6. HRESIMS Spectrum of **1**…………………………………………..……6
Figure S7. UV Spectrum of **1**……………………………………………………….…6
Figure S8. UPLC-MS analysis of the acid hydrolysate of **1**……………………..……7
Figure S9. ^{1}H NMR Spectrum of **2**………………………………………………..…8
Figure S10. ^{13}C and DEPT NMR Spectrum of **2**……………………………..….…8
Figure S11. ^{1}H-^{1}H COSY Spectrum of **2**……………………………………..…9
Figure S12. HSQC Spectrum of **2**……………………..……………..………9
Figure S13. HMBC Spectrum of **2**…………………………………………...…10
Figure S14. NOESY Spectrum of **2**…………………………………..……10
Figure S15. HRESIMS Spectrum of **2**…………………………………..………11
Figure S16. UV Spectrum of **2**……………………………………………….....12
Figure S17. ^{1}H NMR Spectrum of **3**………………………………..………...12
Figure S18. ^{13}C and DEPT NMR Spectrum of **3**……………………….………13
Figure S19. ^{1}H-^{1}H COSY Spectrum of **3**………………………………......…13
Figure S20. HSQC Spectrum of **3**…………………………………..…..…14
Figure S21. HMBC Spectrum of **3**………………………………………...14
Figure S22. NOESY Spectrum of **3**…………………………………..…15
Figure S23. HRESIMS Spectrum of **3**……………………………………..15
Figure S24. UV Spectrum of **3**……………………………………………….....16
The ECD computational detail of compound 3……………………………………16
The physicochemical data of the known compounds…………………………..…17
The strain's (*Aspergillus hiratsukae* SCSIO 5Bn$_1$003) ITS sequence of the rDNA……………………………………………………………………………...20
Table S1. The primary screening of cytotoxic activity of all compounds…………..20
Table S2. The primary screening of antibacterial activity of all compounds……....21
Table S3. The primary screening of antioxidative and α-glucosidase inhibitory activity of all compounds………………………………………………………….....21

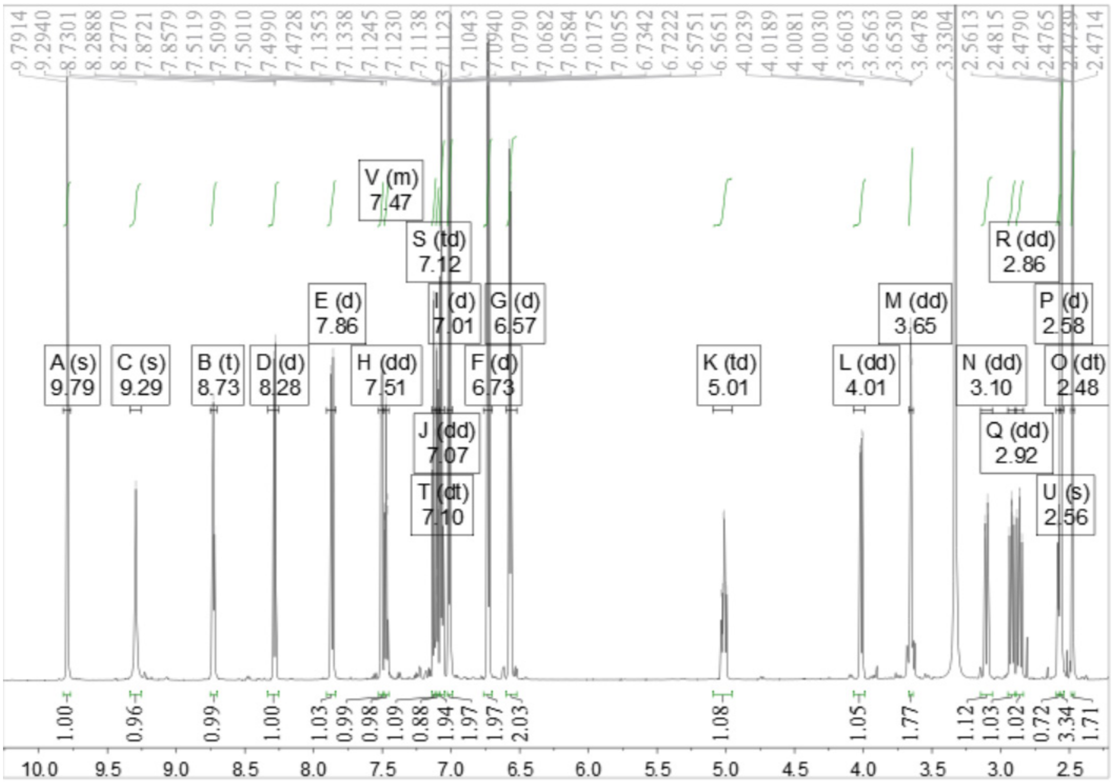

Figure S1. ^{1}H NMR spectrum of asperhiratide **(1)**.

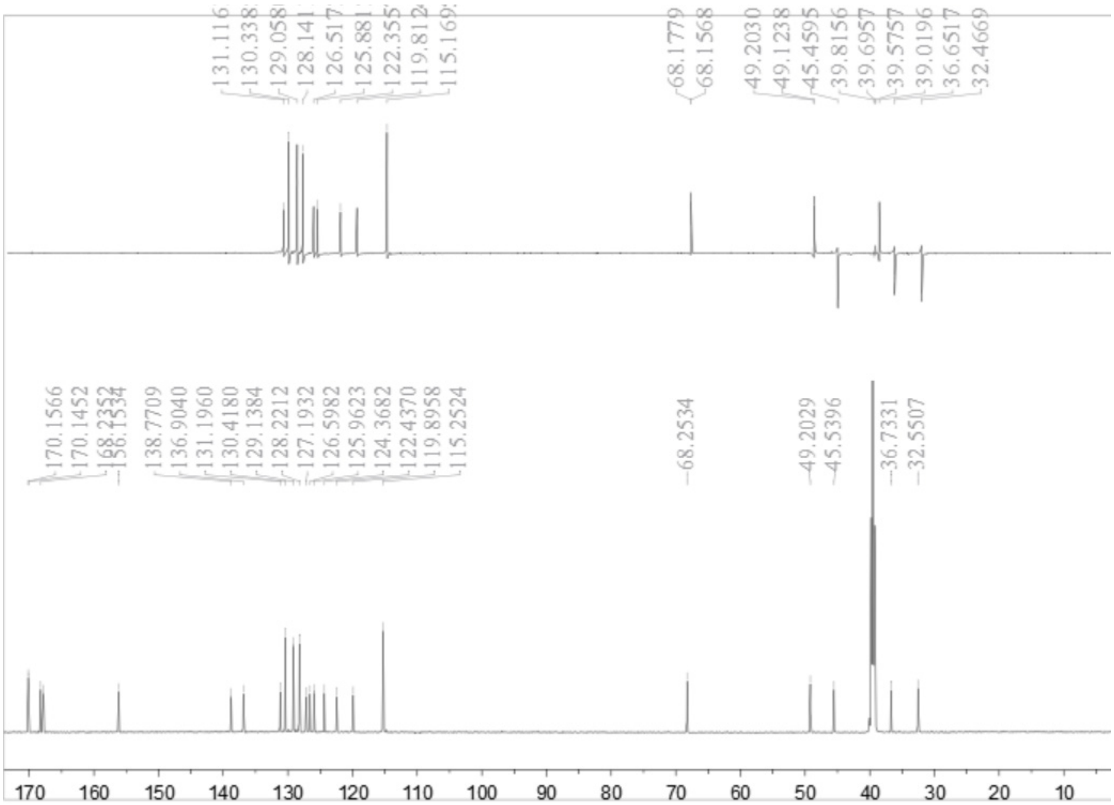

Figure S2. ^{13}C NMR and DEPT spectrum of asperhiratide **(1)**.

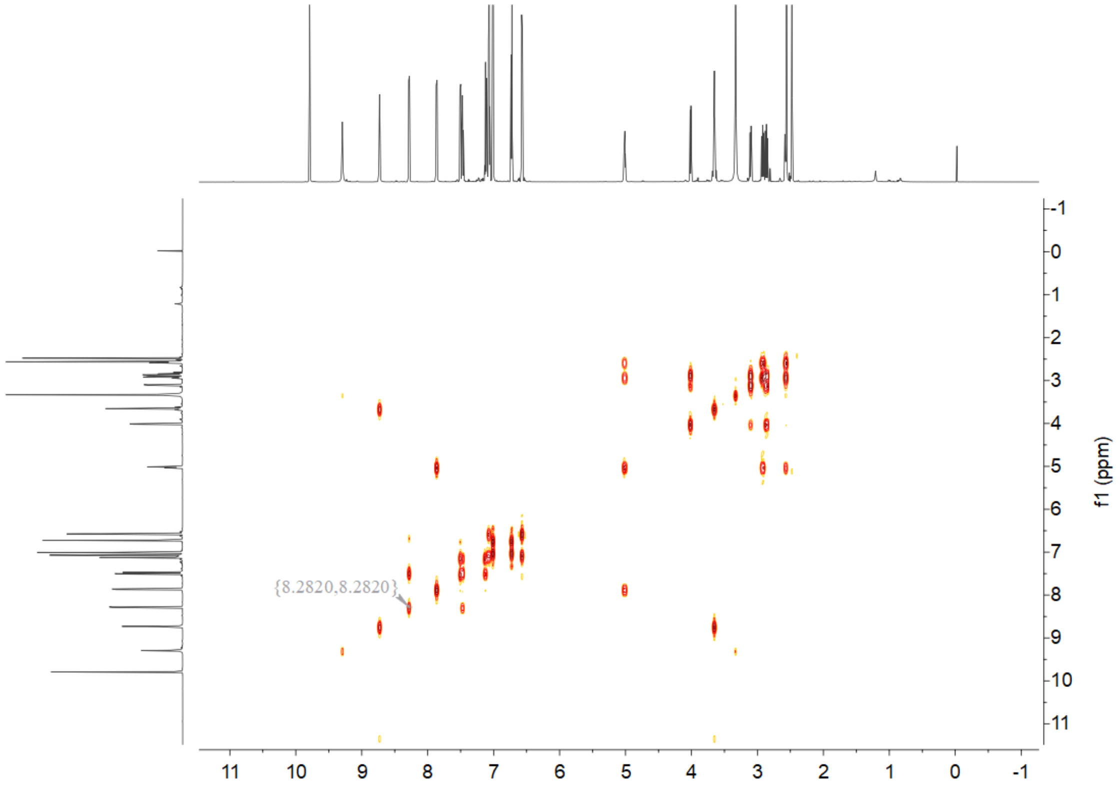

Figure S3. COSY spectrum of asperhiratide **(1)**.

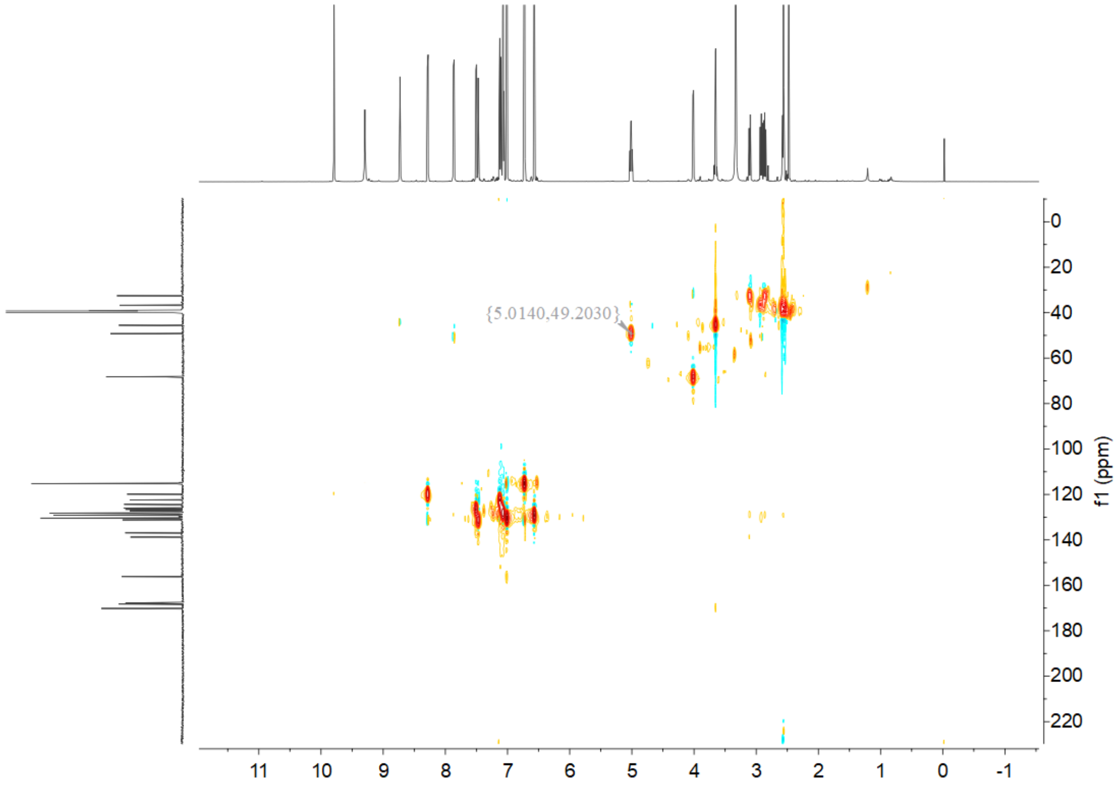

Figure S4. HSQC spectrum of asperhiratide **(1)**.

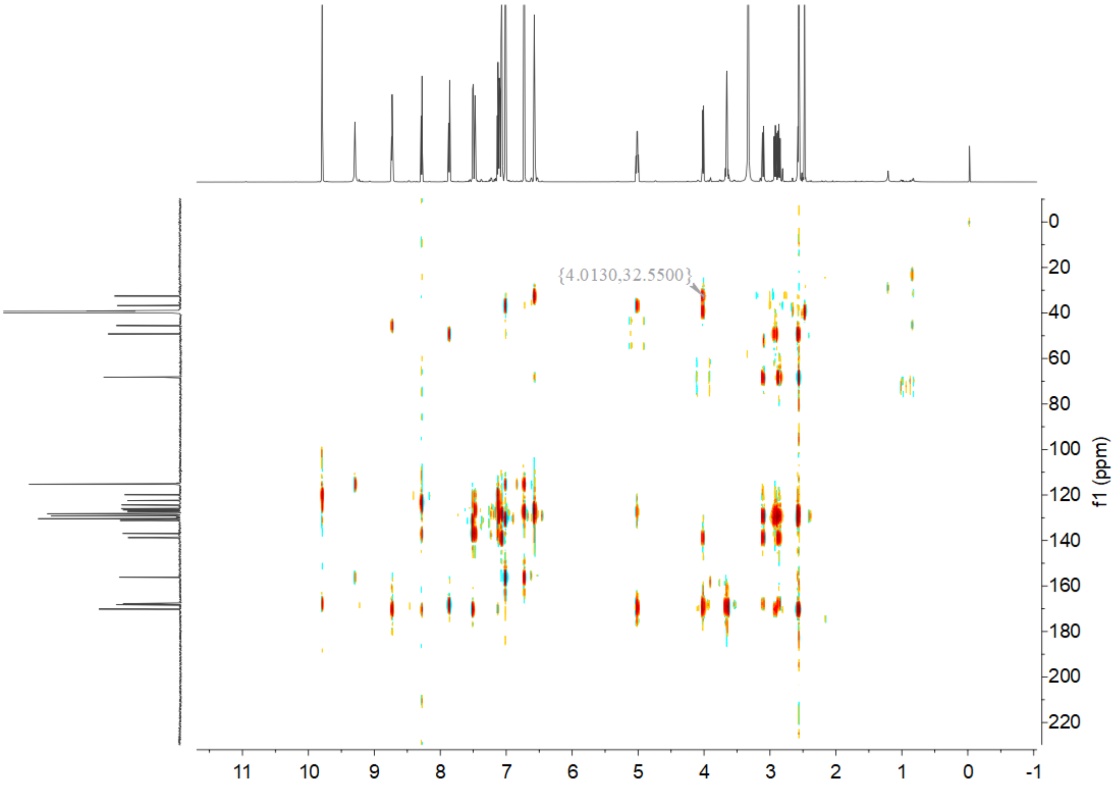

Figure S5. HMBC spectrum of asperhiratide **(1)**.

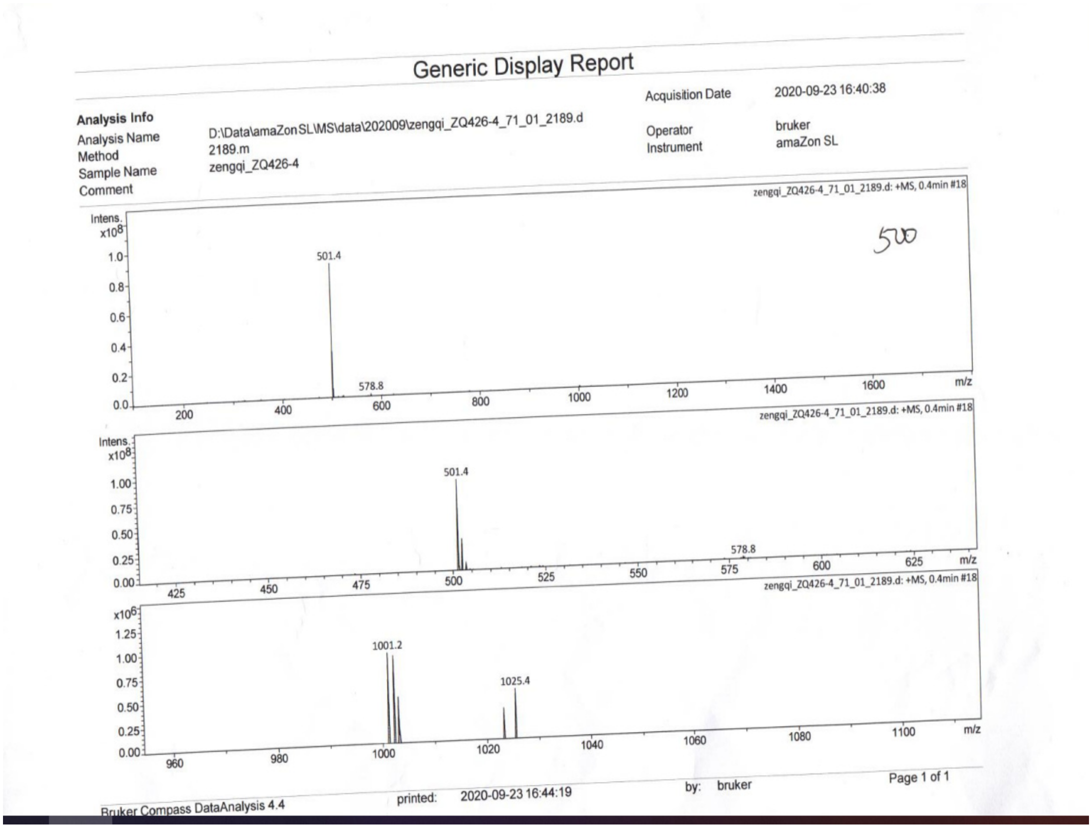

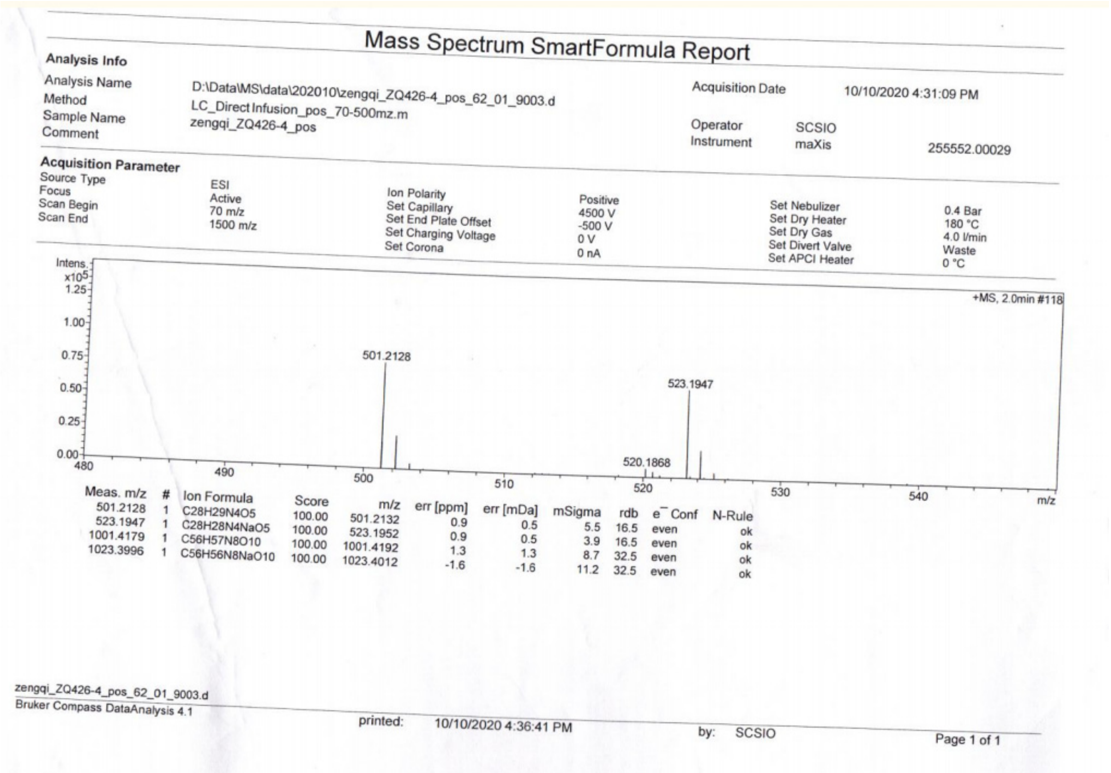

Figure S6. HRESIMS spectrum of asperhiratide **(1)**.

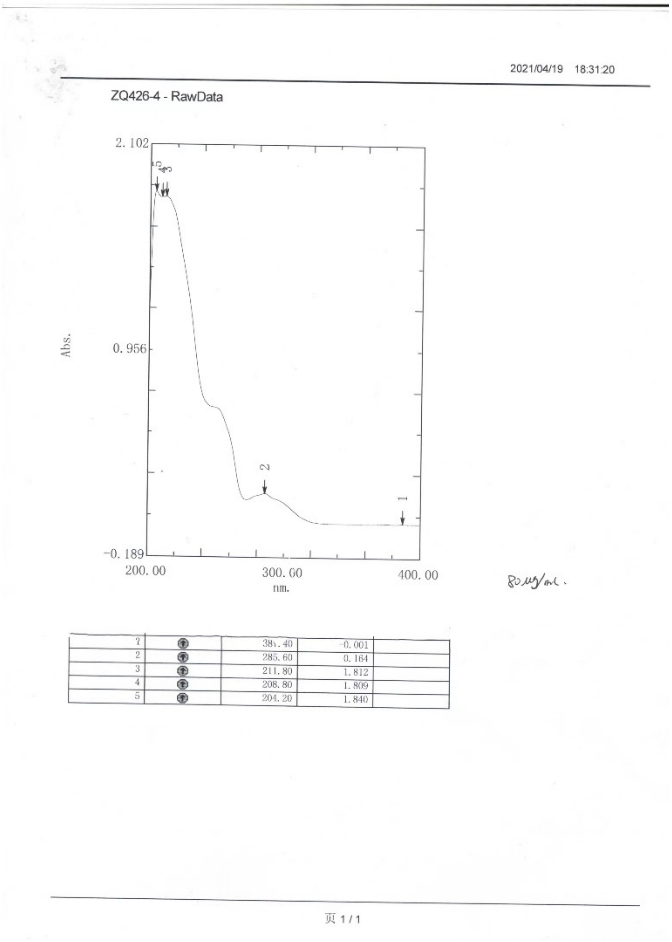

[illegible]		38[illegible].40	-0.001
2		285.60	0.164
3		211.80	1.812
4		208.80	1.809
5		204.20	1.840

Figure S7. UV spectrum of asperhiratide **(1)** (MeOH).

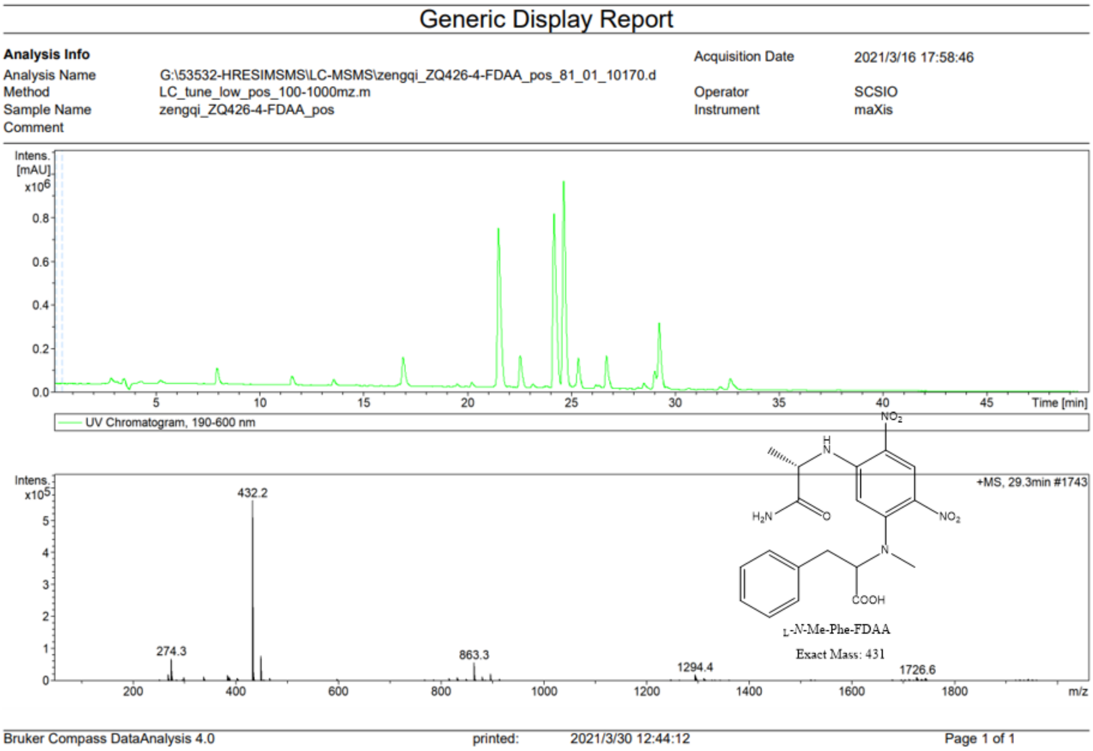

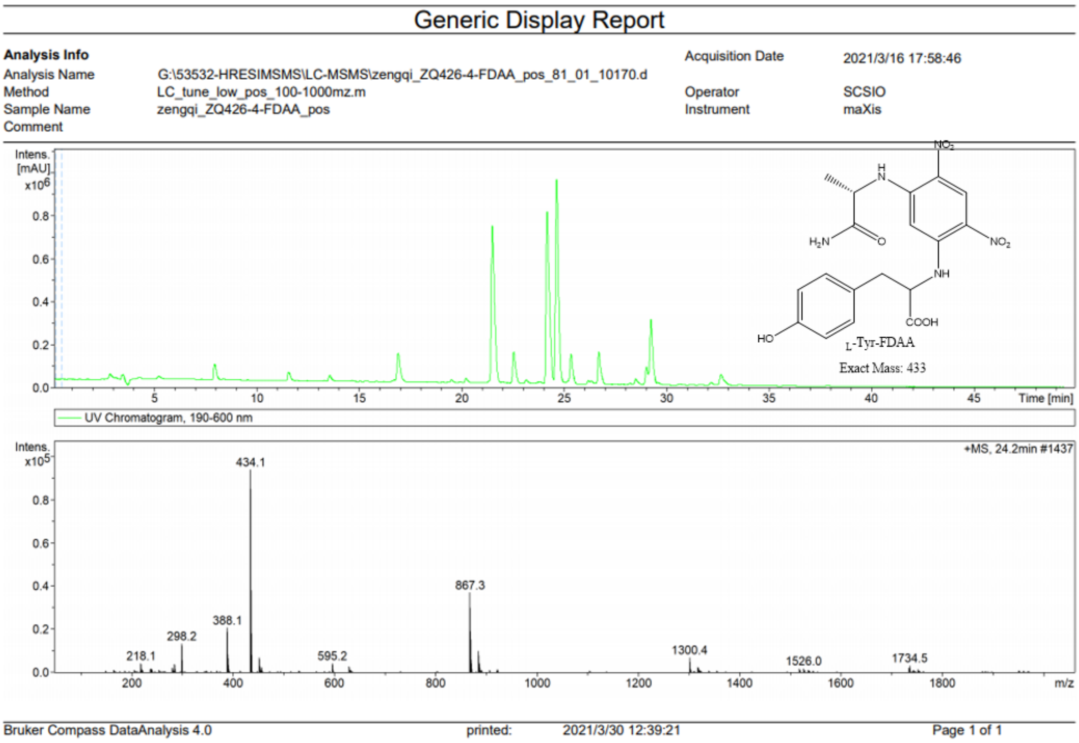

Figure S8. UPLC-MS analysis of the acid hydrolysate of asperhiratide **(1)**.

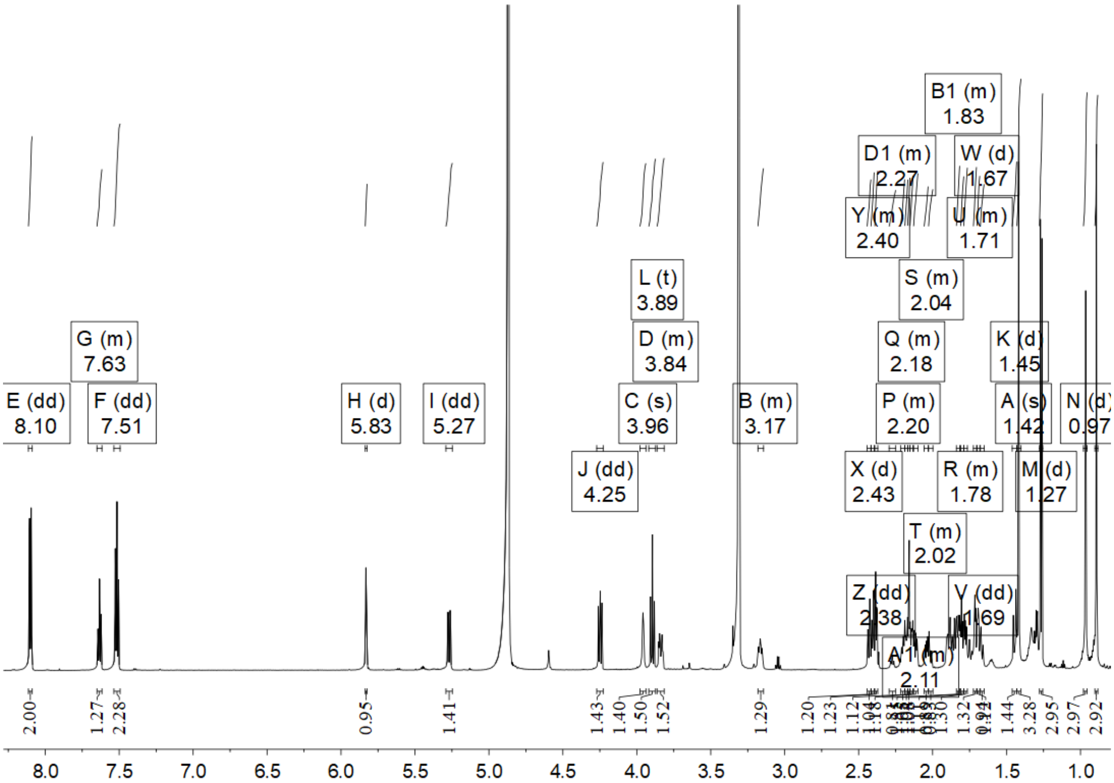

Figure S9. ^{1}H NMR spectrum of asperhiratine **(2)**.

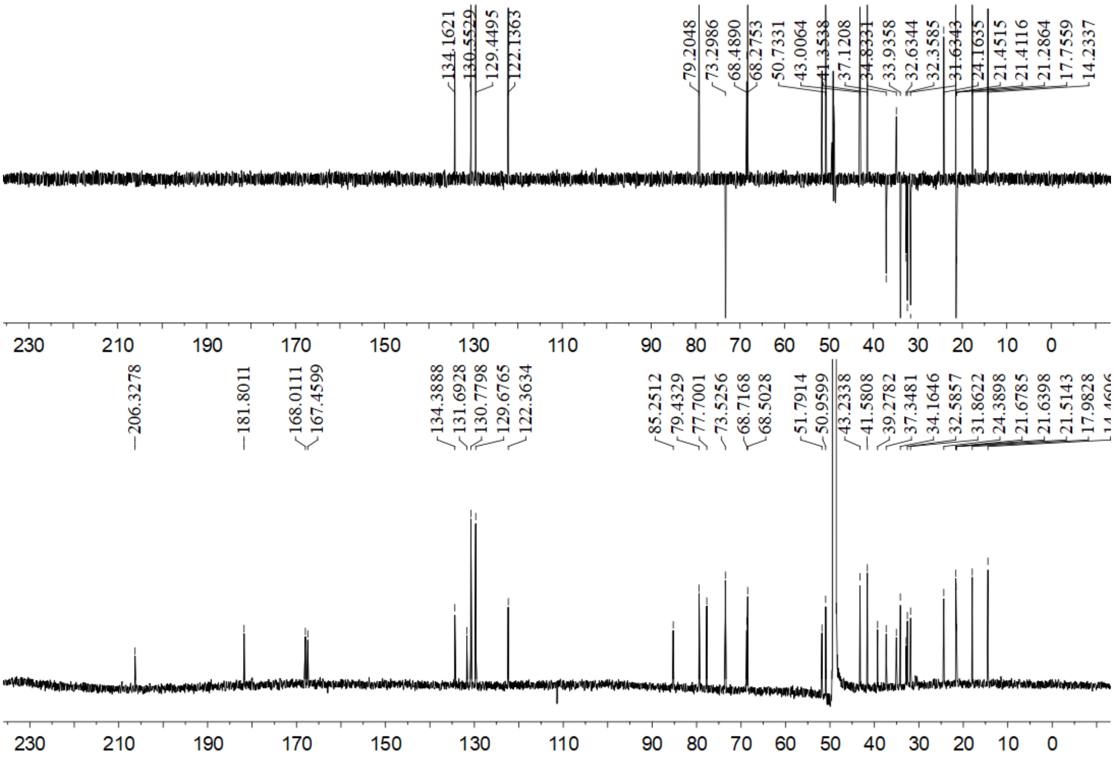

Figure S10. ^{1}C NMR and DEPT spectrum of asperhiratine **(2)**.

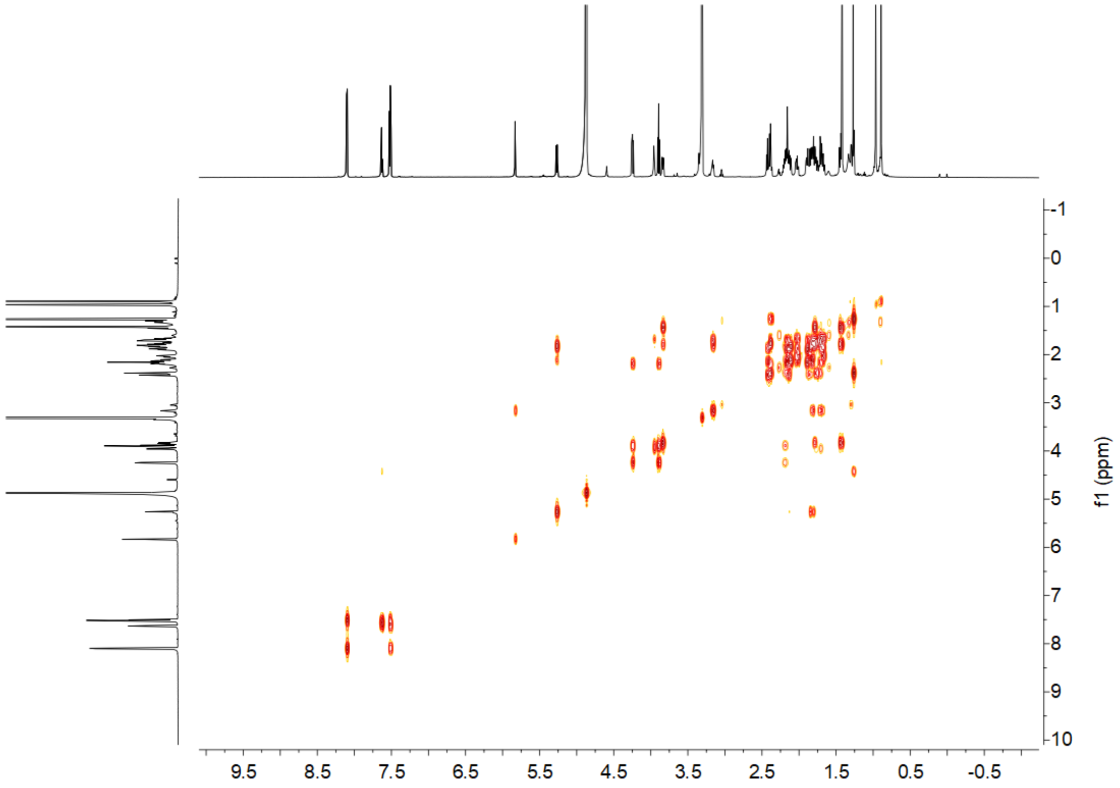

Figure S11. COSY spectrum of asperhiratine **(2)**.

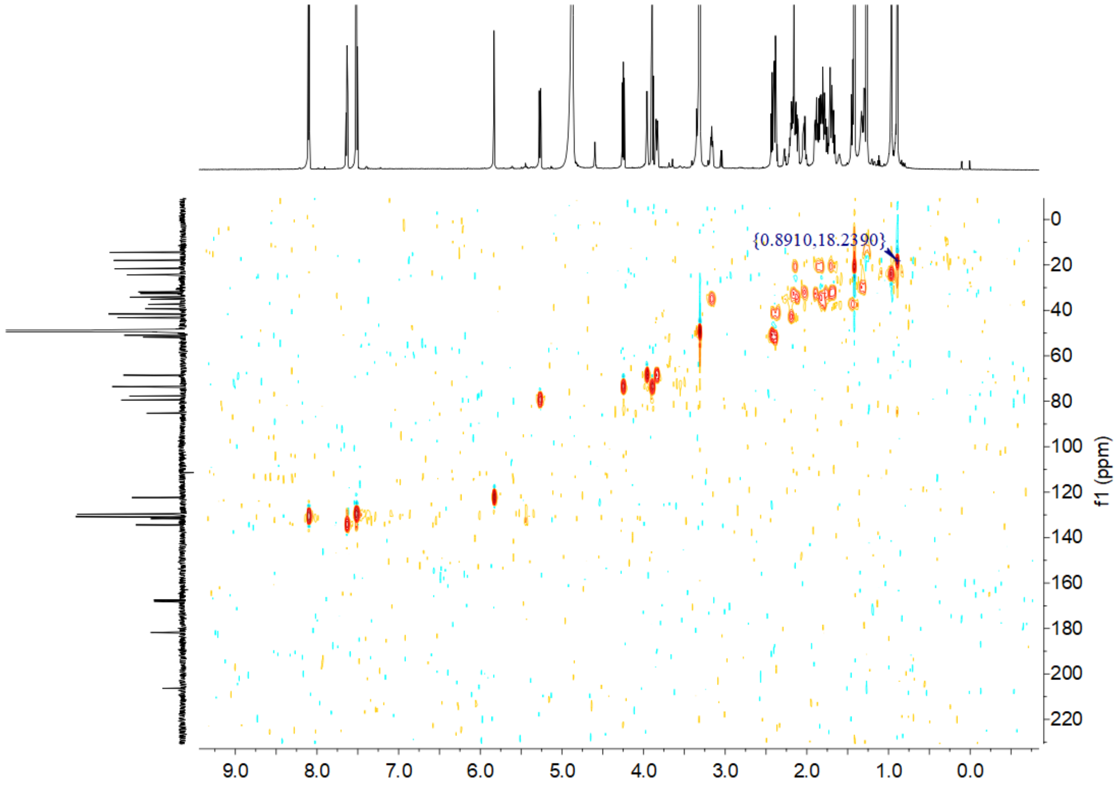

Figure S12. HSQC spectrum of asperhiratine **(2)**.

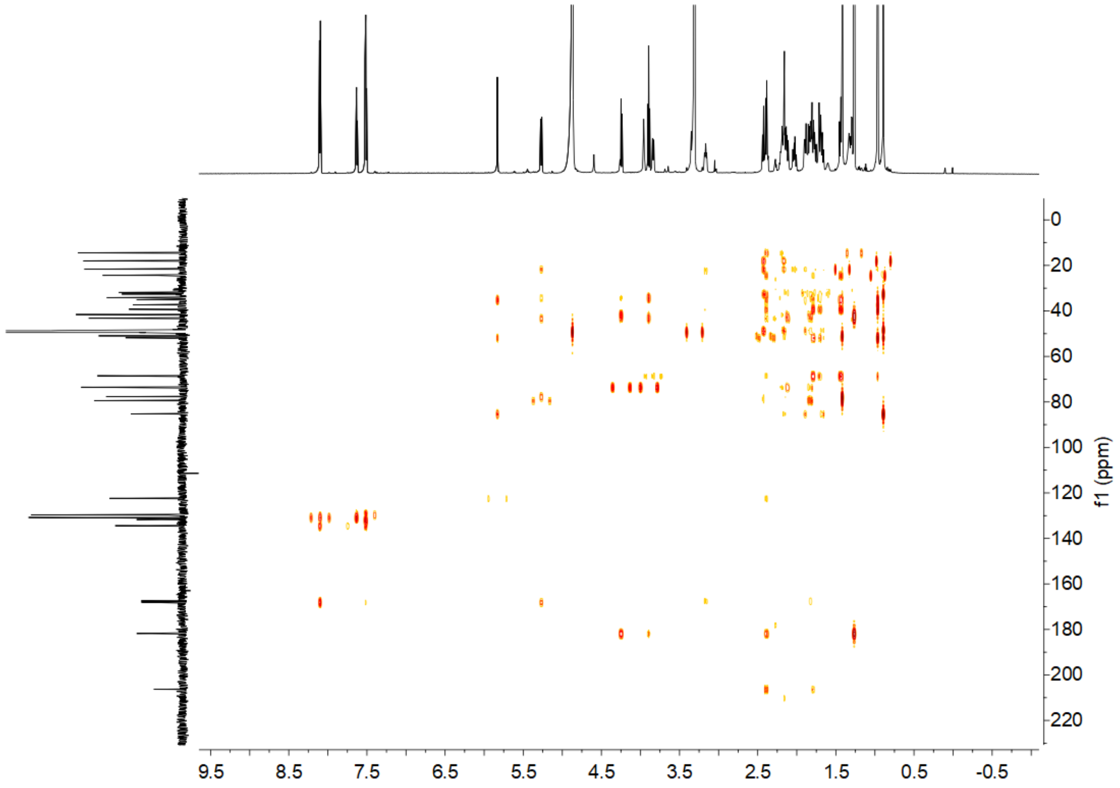

Figure S13. HMBC spectrum of asperhiratine **(2)**.

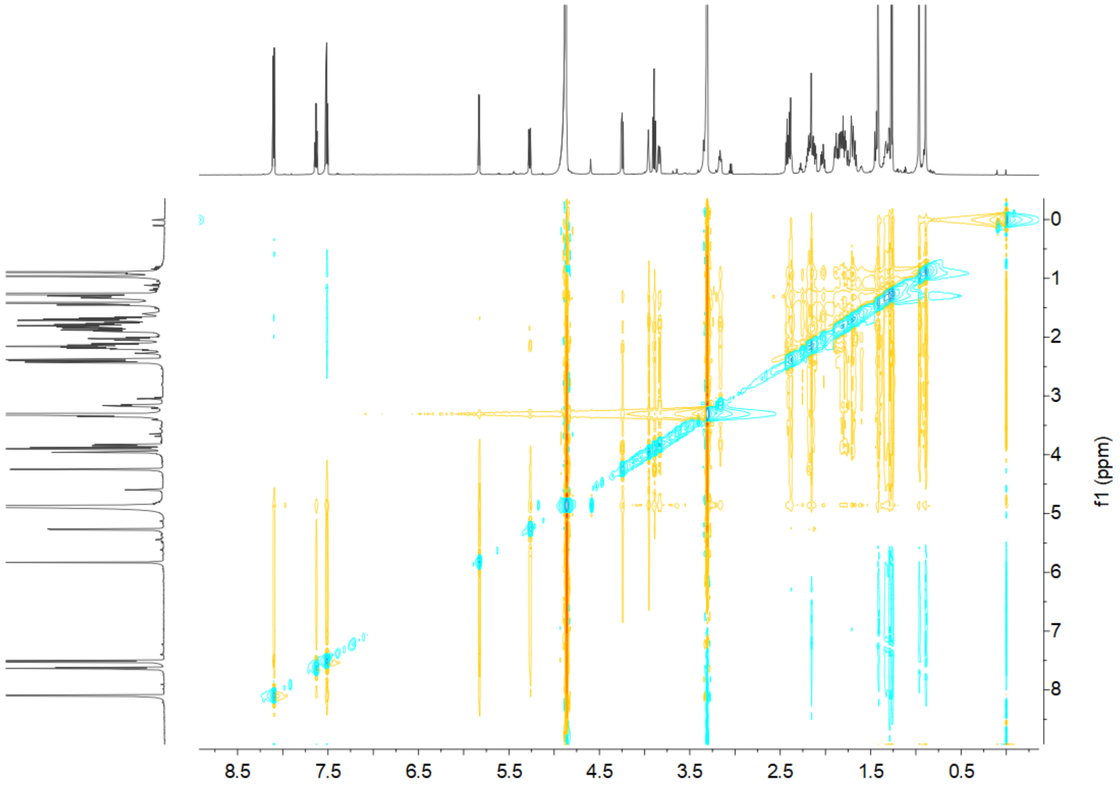

Figure S14. NOESY spectrum of asperhiratine **(2)**.

Generic Display Report

**Analysis Info**		Acquisition Date	12/21/2021 10:07:15 AM
Analysis Name	D:\Data\MS\data\202112\zengqi_ZQ524A_pos_44_01_12125.d		
Method	LC_Direct Infusion_pos_100-1000mz.m	Operator	SCSIO
Sample Name	zengqi_ZQ524A_pos	Instrument	maXis
Comment			

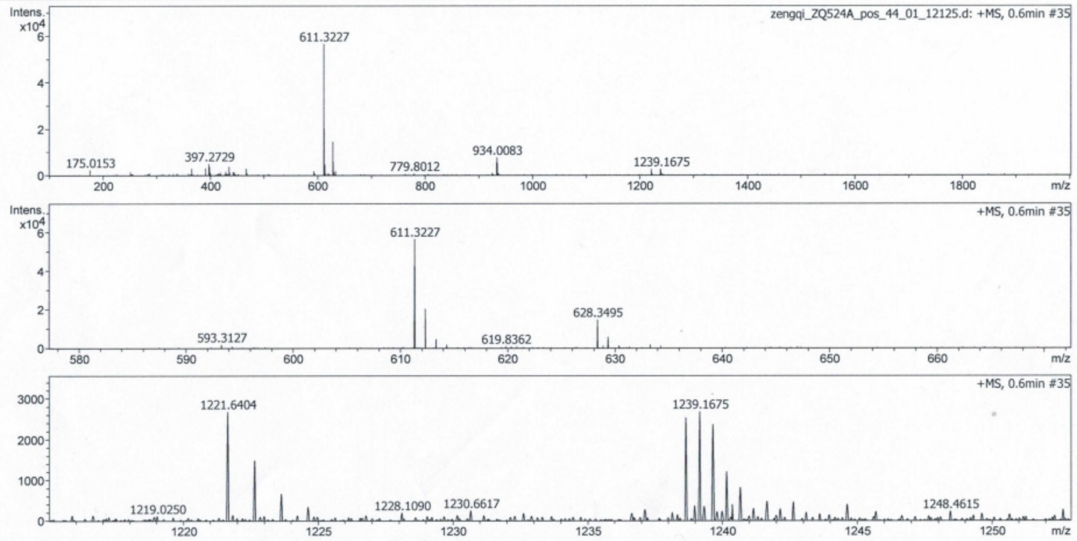

Bruker Compass DataAnalysis 4.1 printed: 12/21/2021 10:14:17 AM by: SCSIO Page 1 of 1

Mass Spectrum SmartFormula Report

**Analysis Info**		Acquisition Date	12/21/2021 10:07:15 AM	
Analysis Name	D:\Data\MS\data\202112\zengqi_ZQ524A_pos_44_01_12125.d			
Method	LC_Direct Infusion_pos_100-1000mz.m	Operator	SCSIO	
Sample Name	zengqi_ZQ524A_pos	Instrument	maXis	255552.00029
Comment				

Acquisition Parameter

Source Type	ESI	Ion Polarity	Positive	Set Nebulizer	0.4 Bar
Focus	Active	Set Capillary	4500 V	Set Dry Heater	180 °C
Scan Begin	100 m/z	Set End Plate Offset	-500 V	Set Dry Gas	4.0 l/min
Scan End	2000 m/z	Set Charging Voltage	0 V	Set Divert Valve	Waste
		Set Corona	0 nA	Set APCI Heater	0 °C

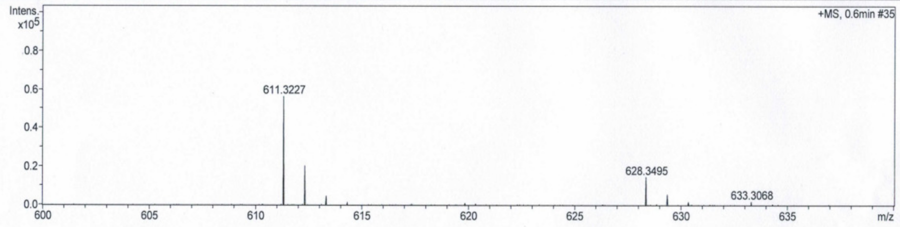

Meas. m/z	#	Ion Formula	Score	m/z	err [ppm]	err [mDa]	mSigma	rdb	e⁻ Conf	N-Rule
611.3227	1	C35H47O9	100.00	611.3215	-2.1	-1.3	14.8	12.5	even	ok
628.3495	1	C35H50NO9	100.00	628.3480	-2.5	-1.5	4.4	11.5	even	ok
1221.6404	1	C70H93O18	100.00	1221.6356	-3.9	-4.7	103.7	24.5	even	ok
1238.6635	1	C70H96NO18	100.00	1238.6622	-1.1	-1.3	74.3	23.5	even	ok

Figure S15. HRESIMS spectrum of asperhiratine **(2)**.

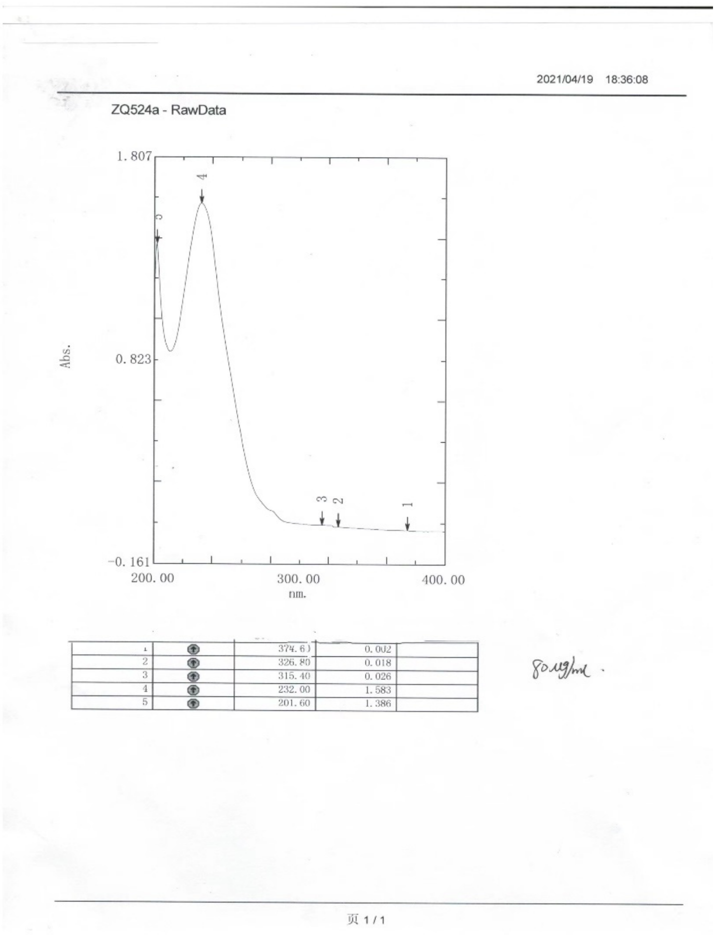

Figure S16. UV spectrum of asperhiratine **(2)**.

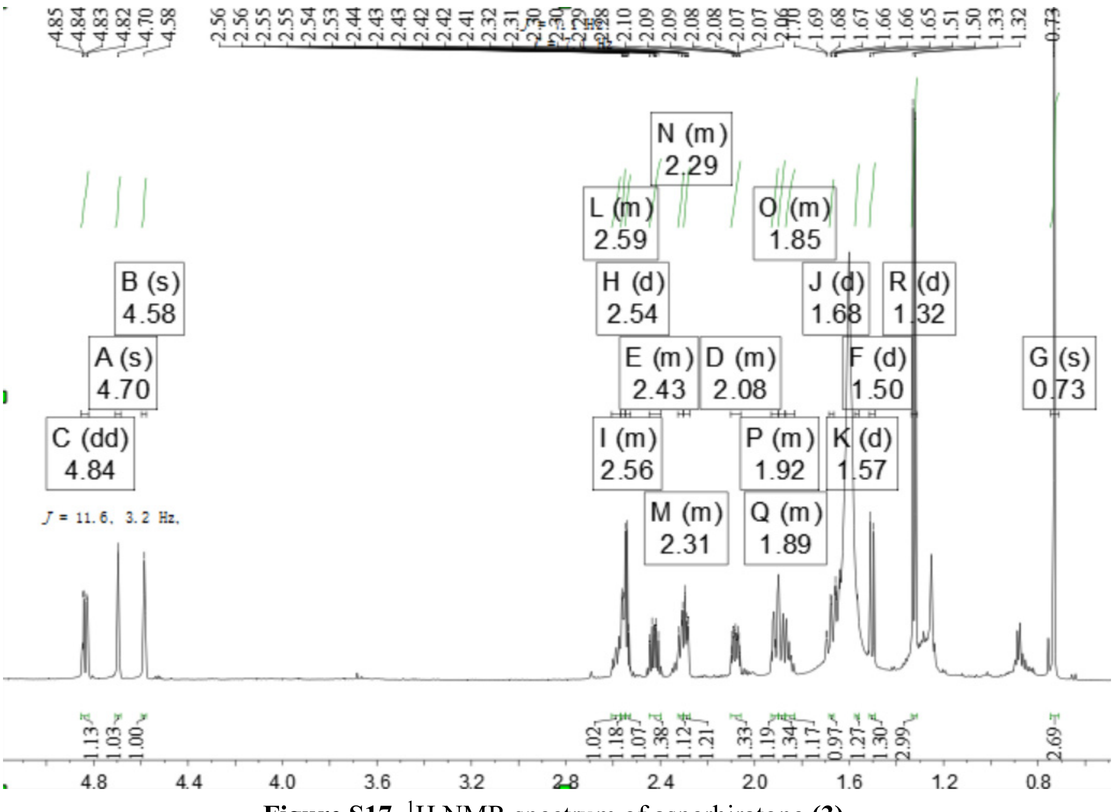

Figure S17. ^{1}H NMR spectrum of asperhiratone **(3)**.

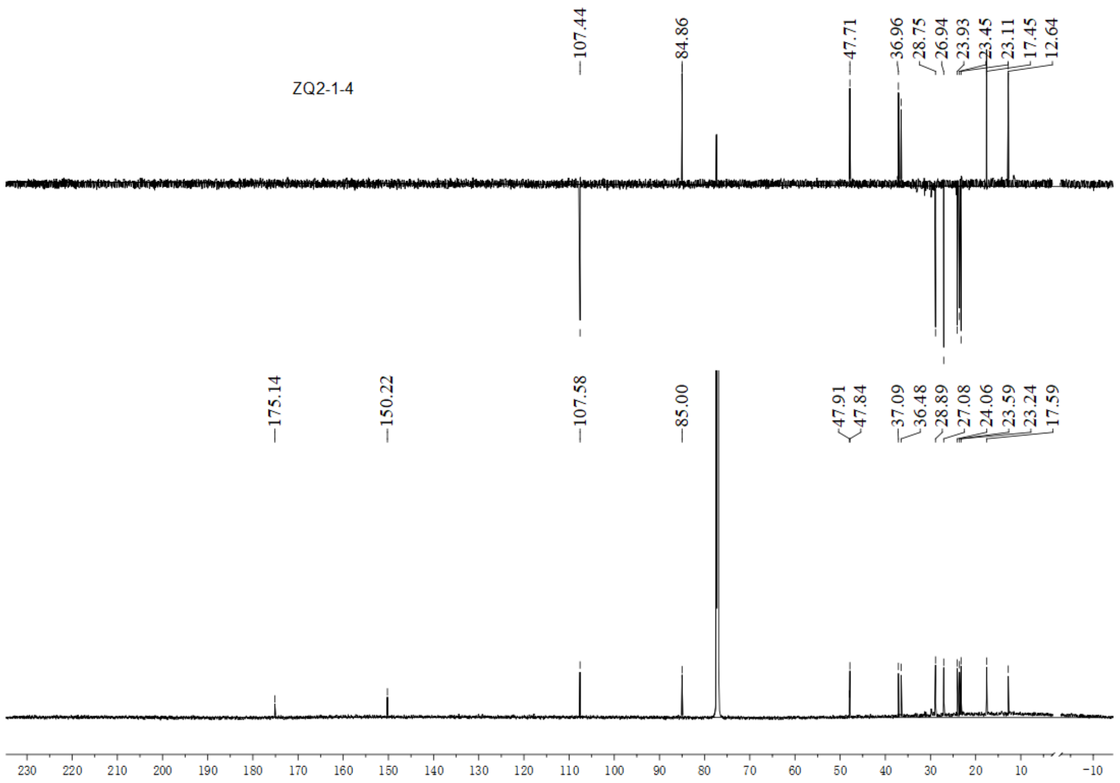

Figure S18. ^{13}C NMR and DEPT spectrum of asperhiratone **(3)**.

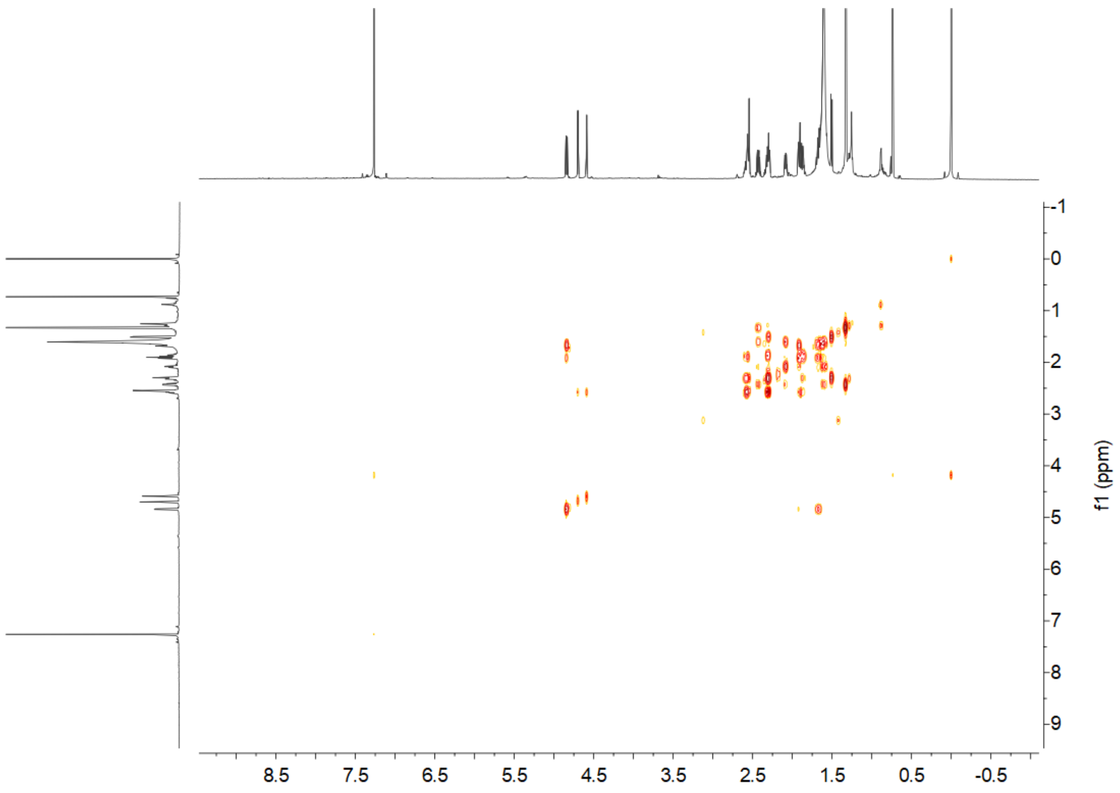

Figure S19. ^{1}H -^{1}H COSY spectrum of asperhiratone **(3)**.

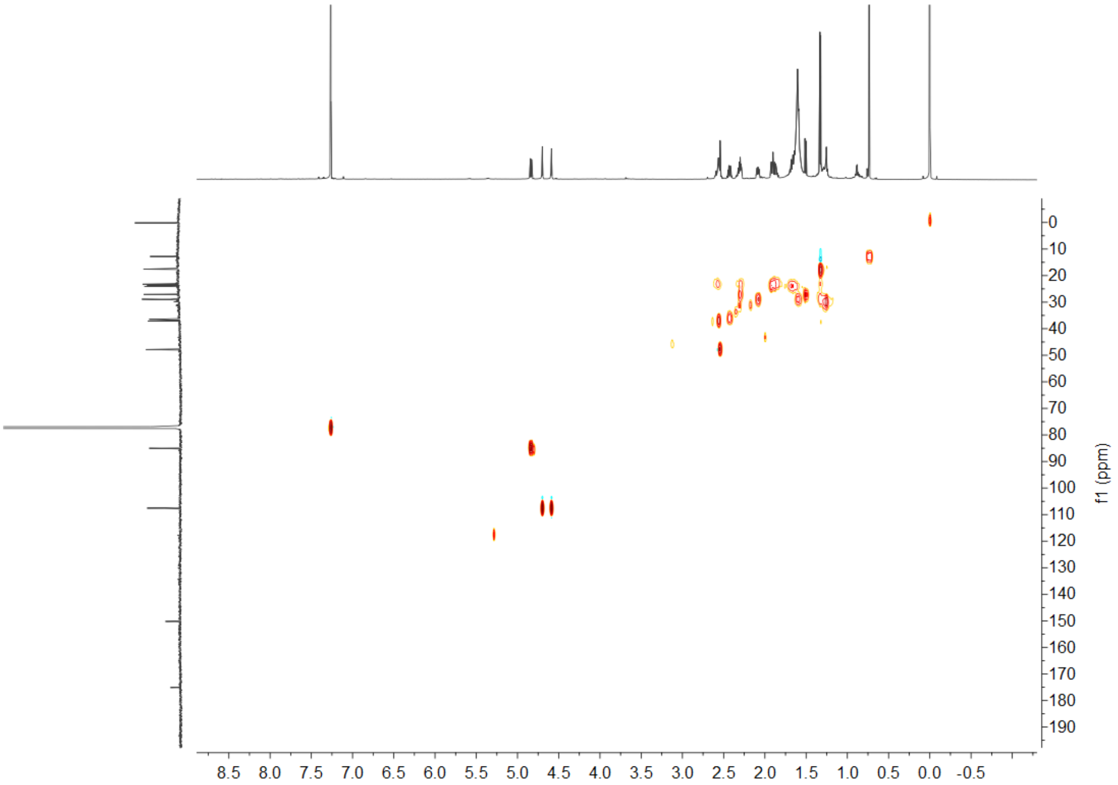

Figure S20. HSQC spectrum of asperhiratone **(3)**.

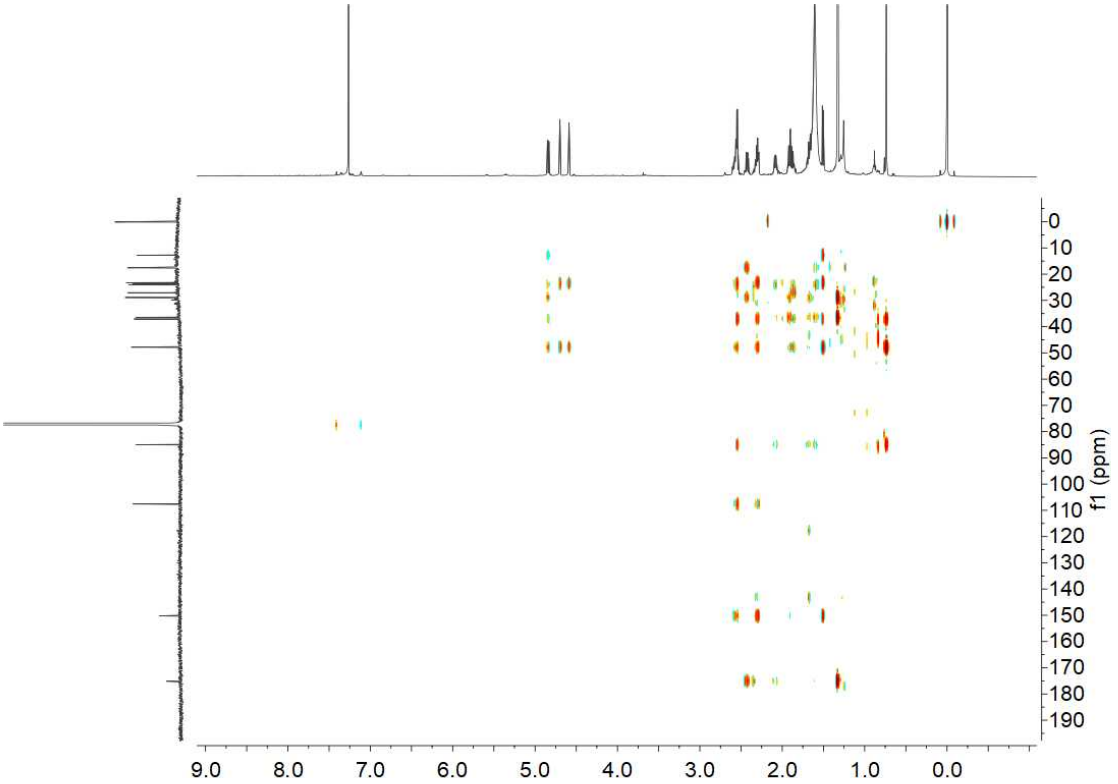

Figure S21. HMBC spectrum of asperhiratone **(3)**.

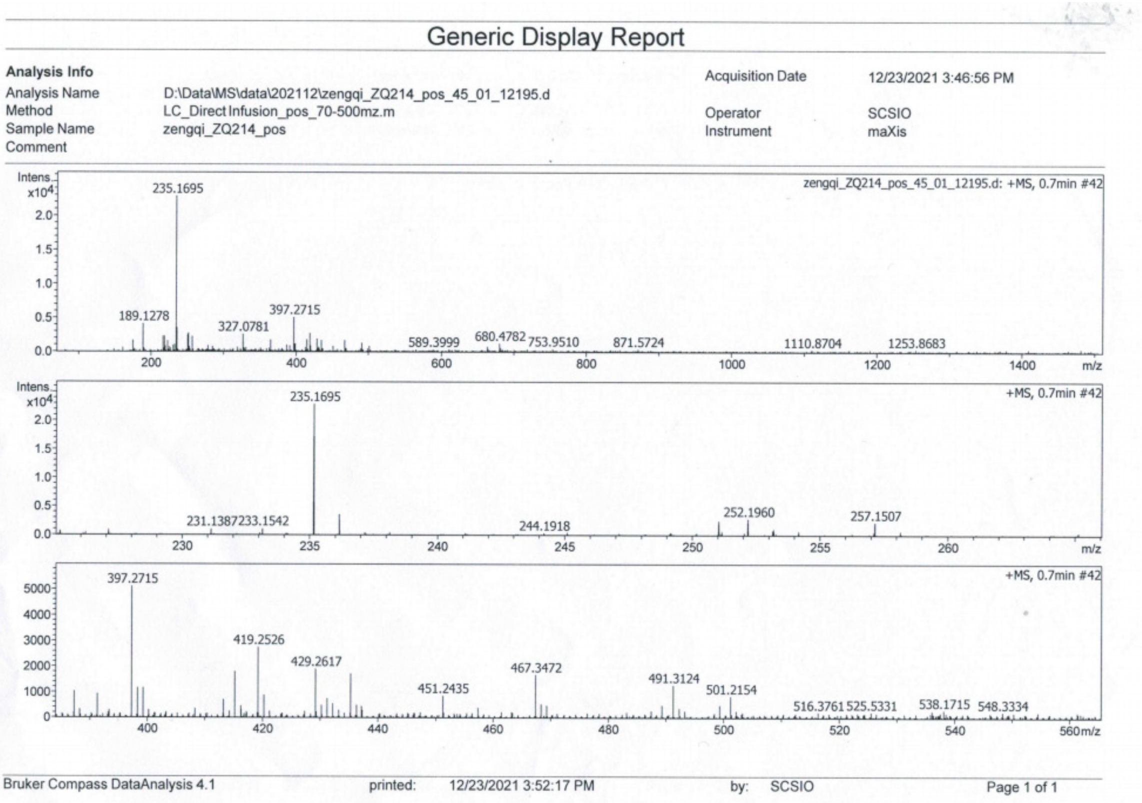

Generic Display Report

Analysis Info

Analysis Name D:\Data\MS\data\202112\zengqi_ZQ214_pos_45_01_12195.d
Method LC_Direct Infusion_pos_70-500mz.m
Sample Name zengqi_ZQ214_pos
Comment

Acquisition Date 12/23/2021 3:46:56 PM
Operator SCSIO
Instrument maXis

Bruker Compass DataAnalysis 4.1 printed: 12/23/2021 3:52:17 PM by: SCSIO Page 1 of 1

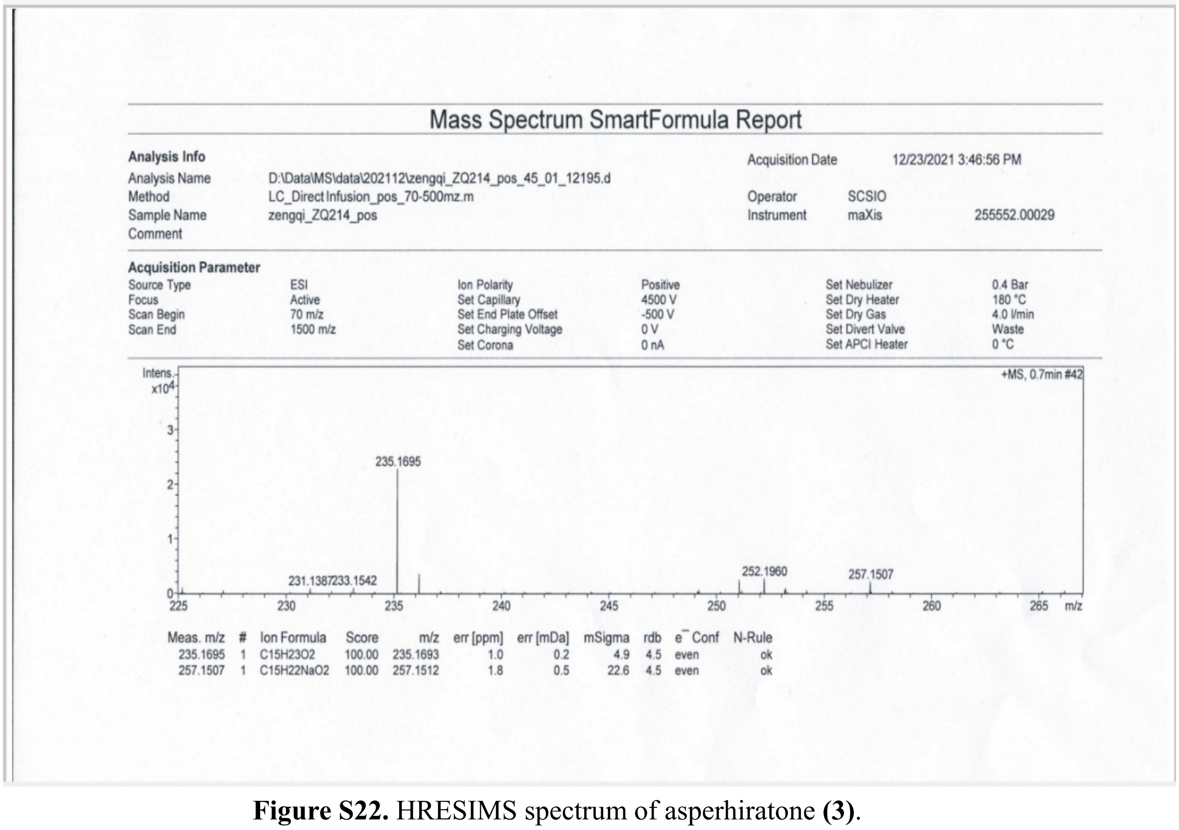

Mass Spectrum SmartFormula Report

Analysis Info

Analysis Name D:\Data\MS\data\202112\zengqi_ZQ214_pos_45_01_12195.d
Method LC_Direct Infusion_pos_70-500mz.m
Sample Name zengqi_ZQ214_pos
Comment

Acquisition Date 12/23/2021 3:46:56 PM
Operator SCSIO
Instrument maXis 255552.00029

Acquisition Parameter

Source Type	ESI	Ion Polarity	Positive	Set Nebulizer	0.4 Bar
Focus	Active	Set Capillary	4500 V	Set Dry Heater	180 °C
Scan Begin	70 m/z	Set End Plate Offset	-500 V	Set Dry Gas	4.0 l/min
Scan End	1500 m/z	Set Charging Voltage	0 V	Set Divert Valve	Waste
		Set Corona	0 nA	Set APCI Heater	0 °C

Meas. m/z	#	Ion Formula	Score	m/z	err [ppm]	err [mDa]	mSigma	rdb	e⁻ Conf	N-Rule
235.1695	1	C15H23O2	100.00	235.1693	1.0	0.2	4.9	4.5	even	ok
257.1507	1	C15H22NaO2	100.00	257.1512	1.8	0.5	22.6	4.5	even	ok

Figure S22. HRESIMS spectrum of asperhiratone **(3)**.

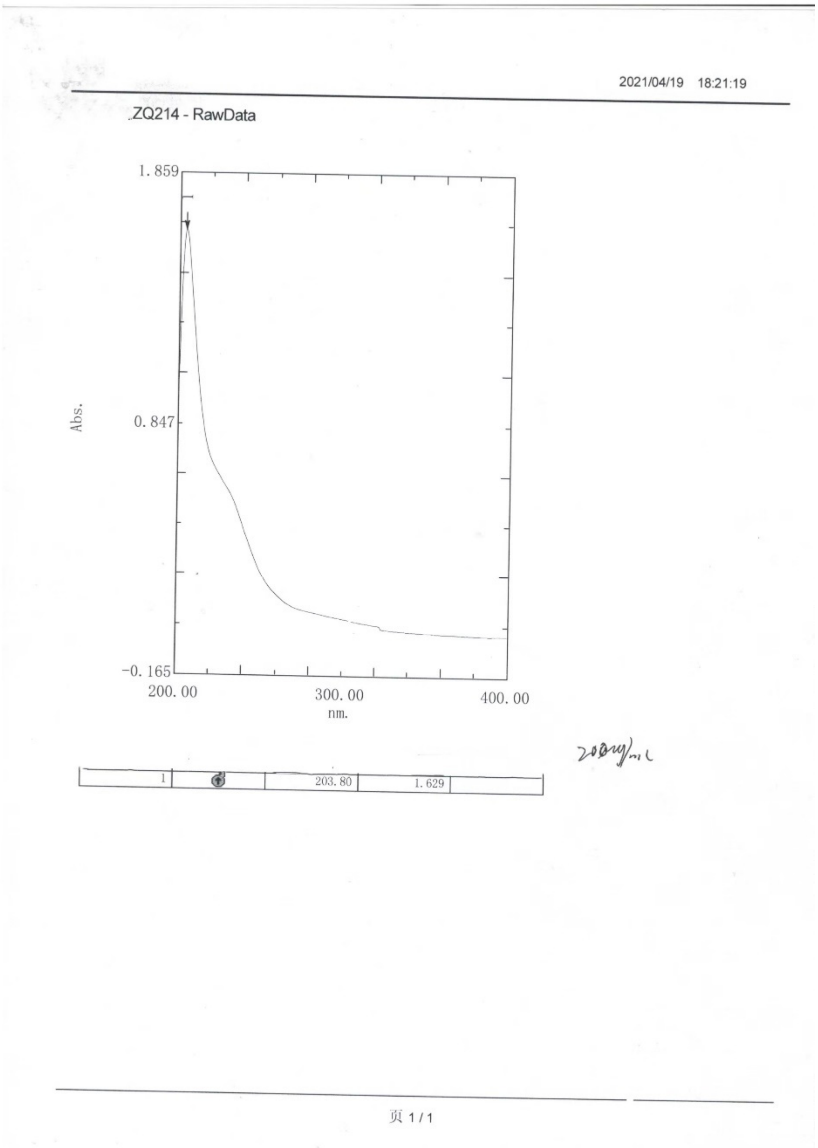

Figure S23. UV spectrum of asperhiratone **(3)**.

The ECD computational detail of compound 3

Conformational searches were done using Molecular Merck force field (MMFF) embedded in Spartan'14 software (Wavefunction Inc., Irvine, CA, USA). Density functional theory (DFT) and time-dependent density functional theory (TDDFT) calculations were performed with Gaussian09 RevD.01. [26]. Double-hybrid (DH) DFT calculations were conducted with ORCA 4.2.1 program package using RIJK approximation, tight SCF criteria, and grid 6 integrity [27]. For simulations of ECD spectrum of compound 3, low-energy conformers of (1R,2S,6S,10R,13S)- and (1R,2S,6S,10S,13R)-3 within a 10 kcal/mol energy window from MMFF conformational search were subjected to geometry optimizations followed by frequency calculations using DFT method at the B3LYP [28,29] /Def2-SVP [30] level of theory with the solvent model PCM for MeOH. The

high level of single point calculations of the optimized low-energy conformers was carried out using the DH-DFT method at the PWPB95 [31] -D3BJ [32,33] / def2-QZVPP[29]. level with the SMD [34] solvent model for MeOH. The TDDFT calculations were carried out using PBE1PBE (PBE0) [35] , M06 [36] and M06-2X [37] functionals in combination with the TZVP [38]. basis set and the PCM solvent (MeOH) model. The number of excited states was 20 for all conformers. The results were visualized and exported using the SpecDis program [39]. The calculated ECD spectra of the two stereoisomers were generated as a sum of Gaussian curve with the σ value of 0.27 eV using rotatory strengths computed in the dipole-velocity gauge from ECD data of the individual low-energy conformers. Boltzmann distributions of the conformers in equilibrium population were estimated from the relative Gibbs free energies (ΔG) at 298.15K.

The physicochemical data of the known compounds

schizaeasterone A (4): white powder; LRESIMS $[M+H]^+$ *m/z* 463.1. $[\alpha]^{25}$D +64.3 (c 0.1 MeOH). ^{1}H NMR (700 MHz, DMSO): δ_H 1.56 (1H, m, H-1a), 1.31 (1H, d, *J*=11.3Hz, H-1b), 1.72 (1H, d, *J*=4.6Hz, H-2a), 1.45 (1H, d, *J*=4.9Hz, H-2b), 3.40 (1H, dd, *J*=10.8, 4.4Hz, H-3), 1.70 (1H, m, H-4a), 1.51(1H, m, H-4b), 1.89 (1H, d, *J*=3.3Hz, H-5), 5.62 (1H, d, *J*=2.4Hz, H-7), 1.87 (1H, d, *J*=4.1Hz, H-9), 1.45 (1H, d, *J*=4.5 Hz , H-11a), 1.56 (1H, m, H-11b) , 1.69 (1H, m, H-12a), 2.20 (1H, m, H-12b), 1.77 (1H, m, H-15a),1.53 (1H, d, *J*=8.7 Hz, H-15b), 1.69 (1H, m, H-16a), 2.20 (1H, m, H-16b), 2.24 (1H, t, *J*=8.7 Hz, H-17), 0.76 (3H, s, H-18), 0.80 (3H, s, H-19), 1.04 (3H, s, H-21), 3.25 (1H, dd, *J*=8.9, 5.4 Hz, H-22), 0.97 (1H, dd, *J*=8.2, 4.1Hz, H-23a), 1.37 (1H, m, H-23b), 1.59 (1H, m, H-24), 1.23 (1H, m, H-25), 0.73 (3H, d, *J*=6.9 Hz, H-26), 0.86(3H, d, *J*=6.9 Hz, H-27), 0.77 (3H, d, *J*=7.0 Hz, H-28), 4.73 (1H, s, OH-3), 4.70 (1H, s, OH-14), 3.60 (1H, s, OH-20), 4.29 (1H, s, OH-22), ^{1}C NMR (700 MHz, DMSO): δ_C 33.2 (CH_2, C-1), δ_C 30.5 (CH_2, C-2), δ_C 67.9 (CH, C-3), δ_C 34.3 (CH_2, C-4), δ_C 55.9 (CH, C-5), δ_C 201.3 (qC, C-6), δ_C 120.2 (CH, C-7), δ_C 165.6 (qC, C-8), δ_C 32.7 (CH, C-9), δ_C 36.0 (qC, C-10), δ_C 19.8 (CH_2, C-11), δ_C 30.9 (CH_2, C-12), δ_C 46.9 (qC, C-13), δ_C 82.9 (qC, C-14), δ_C 30.3 (CH_2, C-15), δ_C 20.3 (CH_2, C-16), δ_C 48.6 (CH, C-17), δ_C 17.1 (CH_3, C-18), δ_C 23.2 (CH_3, C-19), δ_C 75.7 (qC, C-20), δ_C 20.7 (CH_3, C-21), δ_C 73.4 (CH, C-22), δ_C 36.2 (CH_2, C-23), δ_C 34.9 (CH, C-24), δ_C 28.7 (CH, C-25), δ_C 15.8 (CH_3, C-26), δ_C 21.0 (CH_3, C-27), δ_C 15.4 (CH_3, C-28)

terretonin (5): Orange powder; LRESIMS $[M+H]^+$ *m/z* 489.2. $[\alpha]^{25}$D -64.3 (c 0.1 MeOH). ^{1}NMR (700 MHz, DMSO): δ_H 1.80 (1H, dd, *J*=11.6, 6.5 Hz, H-1a), 2.07 (1H, dd, *J*=20.5, 10.3 Hz, H-1b), 2.67 (1H, d, *J*=19.0, 8.7Hz, H-2a), 2.39 (1H, m, H-2b), 2.91 (1H, d, *J*=14.4 Hz , H-11a), 2.29 (1H, d, *J*=14.6 Hz , H-11b) , 3.68 (1H, s, H-14), 1.58 (3H, s, H-20), 1.33 (3H, s, H-21), 1.35 (3H, s, H-24), 1.78 (3H, s, H-26), 4.94 (1H, s, H-27a), 5.17 (1H, s, H-27b), 1.06 (3H, s, H-28), 1.33 (3H, s, H-30), 3.70 (3H, s, H-34). ^{1}C NMR (700 MHz, DMSO): δ_C 32.4 (CH_2, C-1), δ_C 34.0 (CH_2, C-2), δ_C 214.5 (qC, C-3), δ_C 52.0 (qC, C-4), δ_C 130.7 (qC, C-5), δ_C 140.4 (qC, C-6), δ_C 197.1 (qC, C-7), δ_C 43.5 (qC, C-8), δ_C 77.5 (qC, C-9), δ_C 47.2 (qC, C-10), δ_C 27.9 (CH_2, C-11), δ_C 139.1 (qC, C-12), δ_C 48.9 (qC, C-13), δ_C 42.8 (CH, C-14), δ_C 168.0 (qC, C-15), δ_C 84.7 (qC, C-17), δ_C 202.5 (qC, C-18), δ_C 21.2 (CH_3, C-20), δ_C 23.8 (CH_3, C-21), δ_C 21.3 (CH_3, C-24), δ_C 18.6 (CH_3, C-26), δ_C 113.9 (CH_2, C-27), δ_C 18.2 (CH_3, C-28), δ_C 23.1 (CH_3, C-30), δ_C 167.8 (qC, C-31), δ_C 53.5 (CH_3, C-34)

demethylincisterol A2 (6): Orange powder; LRESIMS $[M+H]^+$ *m/z* 333.6. $[\alpha]^{25}$D +10.3 (c 0.2 MeOH). ^{1}H NMR (700 MHz, $CDCl_3$): δ_H 5.64 (1H, d, *J*=1.7 Hz, H-2), δ_H 2.28 (1H, ddd, *J*=14.2,3.9,2.5 Hz, H-5a), δ_H1.86 (1H, m, H-5b), δ_H1.98 (1H, m, H-6a), δ_H1.64 (1H, m, H-6b), δ_H 2.64 (1H, m, H-8), δ_H1.73 (1H, m, H-9a), δ_H1.62 (1H, m, H-9b), δ_H1.92 (1H, m, H-10a), δ_H1.49 (1H, m, H-10b), δ_H 1.50 (1H, m, H-11),δ_H 0.61 (3H, s, H-12), δ_H 2.06 (1H, dd, *J*=15.6, 8.7 Hz, H-13), δ_H 1.04 (3H, d, *J*=6.6 Hz, H-14), δ_H 5.17 (1H, d, *J*=15.3, 8.5 Hz, H-15), δ_H 5.25 (1H, d, *J*=15.2, 7.9 Hz, H-16), δ_H 1.98 (1H, m, H-17), δ_H 1.63 (1H, d, *J*=4.1 Hz, H-18), δ_H 0.84 (3H, d, *J*=6.8 Hz, H-19), δ_H 0.82 (3H, d, *J*=6.7 Hz, H-20), δ_H 0.92 (3H, d, *J*=6.8 Hz, H-21). ^{1}C NMR (500 MHz, $CDCl_3$): δ_C 171.0 (qC, C-1), δ_C 112.5(CH, C-2), δ_C 170.6 (qC, C-3), δ_C 104.9 (qC, C-4),δ_C 35.4 (CH_2, C-5), δ_C 29.0 (CH_2, C-6), δ_C 49.0 (qC, C-7), δ_C 50.5 (CH, C-8), δ_C 21.5 (CH_2, C-9), δ_C 35.1 (CH_2, C-10), δ_C 55.5 (CH, C-11), δ_C 11.9 (CH_3, C-12), δ_C 40.3 (CH, C-13), δ_C 19.8 (CH_3, C-14), δ_C 133.0 (CH, C-15), δ_C 134.8 (CH, C-16), δ_C 42.9 (CH, C-17), δ_C 33.2 (CH, C-18), δ_C 20.1 (CH_3, C-19), δ_C 21.2 (CH_3, C-20), δ_C 17.7 (CH_3, C-21).

asperophiobolin E(7): Orange powder; LRESIMS $[M+H]^+$ *m/z* 383.8. $[\alpha]^{25}$D +86.9 (c 0.2 MeOH);^{1}H NMR (700 MHz, $CDCl_3$): δ_H 1.13 (1H, dd, *J*=22.9, 9.8 Hz, H-1a), δ_H 2.04 (1H, d, *J*=4.2 Hz,H-1b), δ_H 2.84 (1H, d, *J*=12.8 Hz,H-2), δ_H 5.93 (1H, s, H-4), δ_H 3.42 (1H, d, *J*=3.7 Hz, H-6), δ_H 7.03 (1H, d, *J*=4.6 Hz,H-8), δ_H 2.09 (1H, m, H-9a), δ_H 2.75 (1H, d, *J*=19.9 Hz, H-9b), δ_H 2.54 (1H, dd, *J*=10.3, 3.4 Hz, H-10), 1.42 (1H, td, *J*=12.4, 5.1Hz, H-12a), δ_H 1.51 (1H, dt, *J*=14.3, 7.2 Hz, H-12b), δ_H 1.23 (1H,m, H-13a), δ_H 1.65 (1H,m, H-13b), δ_H 1.86 (1H, m, H-14), δ_H 2.56 (1H, dd, *J*=9.9, 3.0 Hz, H-15), δ_H 5.09 (1H, t, *J*=10.1 Hz, H-16), δ_H 6.08 (1H, t, *J*=11.2 Hz, H-17), δ_H 5.99 (1H, t, *J*=11.6Hz, H-18), δ_H 2.07 (3H, s, H-20), δ_H 0.92 (3H, s, H-22), δ_H 0.95 (3H, s, H-23), δ_H 1.75 (3H, s, H-24), δ_H 1.81 (3H, s, H-25). ^{1}C NMR (700 MHz, $CDCl_3$): δ_C 46.2 (CH_2, C-1), δ_C 49.5 (CH, C-2), δ_C 178.6 (qC, C-3), δ_C 129.8 (CH, C-4), δ_C 208.2 (qC, C-5), δ_C 51.4 (CH, C-6), δ_C 127.9 (qC, C-7), δ_C 148.1 (CH, C-8), δ_C 30.1 (CH_2, C-9), δ_C 44.1 (CH, C-10), δ_C 45.5 (qC, C-11), δ_C 44.5 (CH_2, C-12), δ_C 27.9 (CH_2, C-13), δ_C 52.3 (CH, C-14), δ_C 32.6 (CH, C-15), δ_C 135.9 (CH, C-16), δ_C 124.1 (CH, C-17), δ_C 120.1 (CH, C-18), δ_C 136.5 (qC, C-19), δ_C 17.5 (CH_3, C-20), δ_C 17.5 (CH_3, C-20), δ_C 171.6 (qC, C-21), δ_C 23.0 (CH_3, C-22), δ_C 21.4 (CH_3, C-23), δ_C 18.3 (CH_3, C-24), δ_C 26.7 (CH_3, C-25).

butyrolactone I (8): Pale yellow oil; LRESIMS [M-H]− m/z 423.1; [2M-H]− m/z 847.1. [α] D^{25} =+30.5（c 0.8 MeOH）.^{1}H NMR (700 MHz, MeOH): δ_H 3.43 (2H, dd, *J*= 31.1, 14.7Hz, H-5), δ_H 3.79 (3H, s, H-7), δ_H 7.59 (2H, m, H-2'/6'), δ_H 6.85 (2H, m, H-3'/5'), δ_H 6.49 (H, d, *J*= 8.1Hz, H-2''), δ_H 6.41 (H, d, *J*= 2.1Hz, H-5''), δ_H 6.54 (H, dd, *J*= 8.2, 2.2Hz, H-6''), δ_H 3.08 (2H, m, H-7”), δ_H 5.07 (1H, m, H-8”), δ_H 1.67 (3H, s, H-10”), δ_H 1.58 (3H, s, H-11”). ^{1}C NMR (700 MHz, MeOH): δ_C 168.9 (qC, C-1), δ_C 138.3 (qC, C-2), δ_C 127.8 (qC, C-3), δ_C 85.4 (qC, C-4), δ_C 38.2 (CH2, C-5), δ_C 170.2 (qC, C-6), δ_C 52.4 (OCH_3, C-7), δ_C 121.8 (qC, C-1'), δ_C 128.9 (CH, C-2'/6'), δ_C 115.2 (CH, C-3'/5'), δ_C 157.9 (qC, C-4'), δ_C 123.7 (qC, C-1''), δ_C 131.0 (CH, C-2''), δ_C 127.0 (qC, C-3''), δ_C 153.7 (qC, C-4''), δ_C 113.6 (CH, C-5''), δ_C 128.4 (CH, C-6''), δ_C 27.3 (CH_2, C-7”), δ_C 122.2 (CH, C-8”'), δ_C 131.6 (qC, C-9”'), δ_C 24.6 (CH_3, C-10”), δ_C 16.4 (CH_3, C-11”).

butyrolactones VI (9): Colorless solid; LRESIMS $[M+H]^+$ *m/z* 459.8. $[\alpha]^{25}$D +66.8 (c 0.5 MeOH). ^{1}H NMR (700 MHz, MeOH): δ_H 3.45 (2H, d, J=5.7 Hz, H-5), δ_H 3.79 (3H, s, H-7), δ_H 7.58 (2H, m, H-2'/6'), δ_H 6.78 (2H, m, H-3'/5'), δ_H 6.52 (H, d, *J*= 8.1Hz, H-2''), δ_H 6.50 (H, d, J= 2.1Hz, H-5''), δ_H 6.62 (H, dd, J= 8.2, 2.2Hz, H-6''), δ_H 2.69 (1H, dd, *J*= 14.1, 2.1 Hz, H-7”a), δ_H 2.55 (1H, dd, *J*= 14.1, 10.0 Hz, H-7”b), δ_H 3.44(1H, t, *J*= 3.7 Hz, H-8”), δ_H 1.18 (3H, s, H-10”), δ_H 1.18 (3H, s, H-11”). ^{1}C NMR (700 MHz, MeOH): δ_C 168.9 (qC, C-1), δ_C 138.3 (qC, C-2), δ_C 127.8 (qC, C-3), δ_C 85.4 (qC,

C-4), δ_C 38.2 (CH2, C-5), δ_C 170.2 (qC, C-6), δ_C 52.4 (OCH3, C-7), δ_C 121.7 (qC, C-1'), δ_C 129.1 (CH, C-2'/6'), δ_C 115.3 (CH, C-3'/5'), δ_C 157.9 (qC, C-4'), δ_C 124.1 (qC, C-1''), δ_C 132.9 (CH, C-2''), δ_C 127.9 (qC, C-3''), δ_C 154.5 (qC, C-4''), δ_C 114.5 (CH, C-5''), δ_C 129.0 (CH, C-6''), δ_C 32.6 (CH2, C-7”), δ_C 79.2 (CH, C-8”'), δ_C 72.4 (qC, C-9”'), δ_C 24.1 (CH3, C-10”), δ_C 23.7 (CH3, C-11”).

***epi*-aszonalenins B (10)**: White powder; LRESIMS $[M+H]^+$ *m/z* 402.3 $[\alpha]^{25}D$ -3.8 (c 0.1 MeOH) ^{1}H NMR (700 MHz, $CDCl_3$): δ_H 8.03 (1H, s, H-9), δ_H 7.59 (1H, d, *J*=8.0 Hz, H-10), δ_H 7.57 (1H, dd, *J*=8.5, 5.8 Hz, H-11), δ_H 6.89 (1H, d, *J*=5.8 Hz, H-12), δ_H 3.45 (3H, s, H-13), δ_H 1.92 (3H, s, H-14), δ_H 1.92 (3H, s, H-15), δ_H 4.25 (2H, s, H-17), δ_H 2.37 (3H, s, H-19). ^{1}C NMR (500 MHz, $CDCl_3$): δ_C 164.8 (qC, C-2), δ_C 125.6 (qC, C-3), δ_C 126.4 (qC, C-3a), δ_C 125.6 (qC, C-4), δ_C 165.0 (qC, C-5), δ_C 66.0 (qC, C-7), δ_C 152.7 (qC, C-8), δ_C 123.2 (CH, C-9), δ_C 132.3 (qC, C-9a), δ_C 120.2 (CH, C-10), δ_C 131.5 (CH, C-11), δ_C 105.0 (CH, C-12), δ_C 141.6 (qC, C-12a), δ_C 26.5 (CH_3, C-13), δ_C 26.8 (CH_3, C-14), δ_C 26.8 (CH_3, C-15), δ_C 167.8 (qC, C-16), δ_C 54.1 (CH_2, C-17), δ_C 201.7 (qC, C-18), δ_C 30.4 (CH_3, C-20), δ_C 30.4 (CH_3, C-22), δ_C 30.4 (CH_3, C-23), δ_C 30.4 (CH_3, C-24), δ_C 30.4 (CH_3, C-25), δ_C 30.4 (CH_3, C-26).

Flavoglaucin (11): Yellow liquid; LRESIMS $[M+H]^+$ *m/z* 305.2. $[\alpha]^{25}D$ -2.04 (c 0.2 MeOH). ^{1}H NMR (700 MHz, $CDCl_3$): δ_H 10.3 (1H, s, CHO-1), δ_H 6.89 (1H, s, H-4), δ_H 2.88 (1H, m, H-1’), δ_H 1.57 (2H, ddd, *J*=15.6, 8.9, 6.8Hz, H-2’), δ_H 1.39 (2H, qt, *J*=15.2, 7.1Hz, H-3’), δ_H 1.32 (2H, m, H-4’), δ_H 1.27 (2H, m, H-5’), δ_H 1.28 (2H, m, H-6’), δ_H 0.88 (3H, t, *J*=7.1Hz, H-7’), δ_H 3.29 (2H, d, *J*=7.4 Hz, H-1”), δ_H 5.28 (1H, m, H-2”), δ_H 1.76 (3H, s, H-4”), δ_H 1.70 (3H,s, H-5”), δ_H 11.9 (1H, s, OH-2), δ_H 4.47 (1H, s,OH-5). ^{1}C NMR (500 MHz, $CDCl_3$): δ_C 195.6 (CH, CHO-1), δ_C 117.3 (qC, C-1), δ_C 155.8 (qC, C-2), δ_C 128.6 (qC, C-3), δ_C 125.7 (CH, C-4), δ_C 145.0 (qC, C-5), δ_C 128.6 (qC, C-6), δ_C 24.0 (CH_2, C-1’), δ_C 32.0 (CH_2, C-2’), δ_C 29.6 (CH_2, C-3’), δ_C 29.2 (CH_2, C-4’), δ_C 31.8 (CH_2, C-5’), δ_C 22.7 (CH_2, C-6’), δ_C 14.1 (CH_2, C-7’), δ_C 27.0 (CH_2, C-1”), δ_C 121.2 (CH, C-2”), δ_C 133.9 (qC, C-3”), δ_C25.8 (CH_3, C-4”), δ_C 17.8 (CH_3, C-5”).

6,8-Dimethoxy-3-methylisocoumar (12): Orange powder; LRESIMS $[M+H]^+$ *m/z* 223.5. $[\alpha]^{25}D$ -89.3 (c 0.1 MeOH). ^{1}H NMR (700 MHz, MeOH): δ_H 4.45 (1H, ddd, *J*=11.4, 6.3, 2.9 Hz, H-3), δ_H 2.85 (1H, dd, *J*=16.2, 11.4 Hz, H-4), δ_H 2.94 (1H, dd, *J*=16.3, 2.8 Hz, H-4), δ_H 6.55 (1H, d, *J*=2.2Hz, H-5), δ_H 6.48 (1H, d, *J*=2.2 Hz, H-7), δ_H 1.44 (3H, d, *J*=6.3 Hz, H-9), δ_H 3.88 (3H, s, H-10), δ_H 3.88 (3H, s, H-11). ^{1}C NMR (500 MHz, $CDCl_3$): δ_C 164.8 (qC, C-2), δ_C 125.6 (qC, C-3), δ_C 126.4 (qC, C-3a), δ_C 125.6 (qC, C-4), δ_C 165.0 (qC, C-5), δ_C 66.0 (qC, C-7), δ_C 152.7 (qC, C-8), δ_C 123.2 (CH, C-9), δ_C 132.3 (qC, C-9a), δ_C 120.2 (CH, C-10), δ_C 131.5 (CH, C-11), δ_C 105.0 (CH, C-12), δ_C 141.6 (qC, C-12a), δ_C 26.5 (CH_3, C-13), δ_C 26.8 (CH_3, C-14), δ_C 26.8 (CH_3, C-15), δ_C 167.8 (qC, C-16), δ_C 54.1 (CH_2, C-17), δ_C 201.7 (qC, C-18), δ_C 30.4 (CH_3, C-19).

methyl shikimate (13): Yellow liquid; HRESIMS $[M+H]^+$ *m/z* 189.1560. $[\alpha]^{25}D$ -1.38 (c 0.2 MeOH) ^{1}H NMR (700 MHz, DMSO): δ_H 6.48 (1H, m, H-2), δ_H 4.17 (1H, d, *J*=1.6 Hz, H-3), δ_H 3.74 (1H, s, H-4), δ_H3.61 (1H, m, H-5), δ_H 2.15 (1H, m, H-6a),δ_H2.29 (1H, dd, *J*=16.8, 5.8 Hz, H-6b), δ_H 3.65 (3H, s, H-8), δ_H 4.87 (1H, d, *J*=7.4 Hz, OH-3), δ_H 4.58 (1H, d, *J*=3.5 Hz, OH-4), δ_H 4.76 (1H, d, *J*=5.4 Hz, OH-5),^{1}C NMR (700 MHz, DMSO): δ_C 127.8 (qC, C-1), δ_C 140.8 (CH, C-2), δ_C 68.3 (CH, C-3), δ_C 70.9 (CH, C-4), δ_C 67.6 (CH, C-5), δ_C 29.0 (CH_2, C-6), δ_C 166.7 (qC, C-7),δ_C 51.8 (CH_3, C-8).

The strain’s (*Aspergillus hiratsukae* SCSIO 5Bn$_1$003) ITS sequence of the rDNA

TCAACCTCCCACCCGTGTCTATTGTACCTTGTTGCTTCGGCGGGCCCGCCG
TTTTCGAACGGCCGCCGGGGAGGCCTCGCGCCCCCGGGCCCGCGCCCGCC

GAAGACCCCAACATGAACGCTGTTCTGAAAGTATGCAGTCTGAGTTTGAT
TATCATAATCAGTTAAAACTTTCAACAACGGATCTCTTGGTTCCGGCATCG
ATGAAGAACGCAGCGAAATGCGATAAGTAATGTGAATTGCAGAATTCAGT
GAATCATCGAGTCTTTGAACGCACATTGCGCCCCCTGGTATTCCGGGGGG
CATGCCTGTCCGAGCGTCATTGCTGCCCTCAAGCACGGCTTGTGTGTTGGG
CCCCCGTCCCCGGTTCTCCCCGGGGACGGGCCCGAAAGGCAGCGGCGGCA
CCGCGTCCGATCCTCGAGCGTATGGGGCTTTGTCACCCGCTCTGTAGGCCC
GGCCGGCGCCAGCCGACACCCCAACTTTATTTTTCTAAGGTTGACCTCGGA
TCAGGTAGGGATACCCGCTGAACTTAAGCATATCAATAA

Table S1. the primary screening of cytotoxic activity of all compounds.

compounds	Inhibition rate (50 μM) %			
	SF-268	MCF-7	HepG-2	A549
**1**	0.82	1.03	0.38	4.40
**2**	1.43	1.01	1.45	1.21
**3**	2.31	3.24	0.13	0.83
**4**	8.79	19.08	1.57	8.60
**5**	4.58	5.62	3.52	2.97
**6**	93.24	92.20	81.44	0.41
**7**	9.64	38.75	40.71	0.58
**8**	41.92	50.70	69.11	21.53
**9**	8.47	17.74	5.43	0.29
**10**	32.90	42.17	6.70	0.73
**11**	0.81	0.49	0.39	1.53
**12**	4.43	6.09	2.34	2.91
**13**	0.59	0.66	0.66	0.36

Table S2. the primary screening of antibacterial activities of all compounds.

compounds	Inhibition rate (50 μM) %	
	Staphylococcus aureus	*Bacillus subtilis*
**1**	5.22	3.87
**2**	4.34	0.67
**3**	7.98	9.17
**4**	19.38	5.23
**5**	11.80	8.18
**6**	29.44	79.64
**7**	82.36	94.33
**8**	84.36	91.28
**9**	19.65	3.76
**10**	12.62	0.30
**11**	12.97	17.50
**12**	9.43	2.16
**13**	11.78	7.69

Table S3. the primary screening of antioxidative and *α*-glucosidase inhibitory activities of all compounds.

compounds	Inhibition rate (100 μM) %	
	antioxidative activity	*α*-Glucosidase Inhibitory
**1**	4.30	40.90
**2**	1.60	31.00
**3**	2.00	22.00
**4**	2.80	14.40
**5**	1.90	14.40
**6**	1.00	77.90
**7**	3.60	5.00
**8**	2.50	73.30
**9**	0.60	37.90
**10**	0.40	39.30
**11**	18.20	18.00
**12**	5.50	28.30
**13**	32.90	9.00
